# Transforming growth factor beta induces sensory neuronal hyperexcitability, and contributes to pancreatic pain and hyperalgesia in rats with chronic pancreatitis

**DOI:** 10.1186/1744-8069-8-65

**Published:** 2012-09-11

**Authors:** Yaohui Zhu, Tugba Colak, Mohan Shenoy, Liansheng Liu, Kshama Mehta, Reetesh Pai, Bende Zou, Xinmin Simon Xie, Pankaj J Pasricha

**Affiliations:** 1 Johns Hopkins Center for Neurogastroenterology, Department of Medicine, Division of Gastroenterology and Hepatology, Baltimore, MD, 21205, USA; 2 Department of Medicine, Stanford University School of Medicine, Stanford, CA, USA; 3 Afasci Inc, Redwood City, CA, USA; 4 Department of Pathology, University of Pittsburgh, Pittsburgh, PA, USA

**Keywords:** Transforming growth factor beta, Chronic pain, Neuronal sensitization, Kv channels, Sensory neurons, Chronic pancreatitis

## Abstract

**Background:**

Transforming growth factor beta (TGFβ) is upregulated in chronic inflammation, where it plays a key role in wound healing and promoting fibrosis. However, little is known about the peripheral effects of TGFβ on nociception.

**Methods:**

We tested the in vitro effects of TGFβ1 on the excitability of dorsal root ganglia (DRG) neurons and the function of potassium (K) channels. We also studied the effects of TGFβ1 infusion on pain responses to noxious electrical stimulation in healthy rats as well as the effects of neutralization of TGFβ1 on evoked pain behaviors in a rat model of chronic pancreatitis.

**Results:**

Exposure to TGFβ1 in vitro increased sensory neuronal excitability, decreased voltage-gated A-type K^+^ currents (IA) and downregulated expression of the Kv1.4 (KCNA4) gene. Further TGFβ1 infusion into the naïve rat pancreas in vivo induces hyperalgesia and conversely, neutralization of TGFβ1 attenuates hyperalgesia only in rats with experimental chronic pancreatitis. Paradoxically, TGFβ1 neutralization in naïve rats results in pancreatic hyperalgesia.

**Conclusions:**

TGFβ1 is an important and complex modulator of sensory neuronal function in chronic inflammation, providing a link between fibrosis and nociception and is a potentially novel target for the treatment of persistent pain associated with chronic pancreatitis.

## Background

Sustained/chronic sensitization of sensory neurons, resulting in pathological pain, can be induced by various components of the inflammatory milieu including physico-chemical factors (temperature, acid) as well as a variety of small molecules, cytokines, growth factors, other peptides and enzymes that are a hallmark of chronic inflammation [1]. Transforming growth factor beta (TGFβ) is also prominently expressed in such situations and plays a key role in wound healing and promoting fibrosis. TGFβ and other members of its superfamily including activin and bone morphogenetic proteins (BMP) are recognized as playing critical roles in the development, survival and repair of neurons in the peripheral and central nervous systems (CNS) [2,3]. Intact and injured dorsal root ganglia (DRG) neurons produce TGFβ and express TGFβ receptors [4,5], and endogenous TGF potentiates the trophic effect of other growth factors on DRG neurons [6,7]. Despite this knowledge, the role of TGFβ on peripheral noccieptor sensitization remains unknown.

We hypothesized that TGFβ is an important modulator of peripheral sensory neuronal function and plays a major role in the pathogenesis of pain in chronic inflammatory disorders. We tested this hypothesis in chronic pancreatitis, as TGFβ is known to be upregulated in the pancreas in this condition in rodents as well as humans [8,9]. Here we show that TGFβ1 can directly sensitize nociceptors, induce pancreatic hyperalgesia and contribute to the enhanced nocifensive response that accompanies chronic pancreatitis. These changes are accompanied by a decrease in IA voltage-dependent potassium currents and downregulation of the KCNA4 gene that may encode this function, providing a possible mechanistic explanation for these findings.

## Results

### TGFβ1 sensitizes nociceptor neurons in vitro

**Figure 1:**
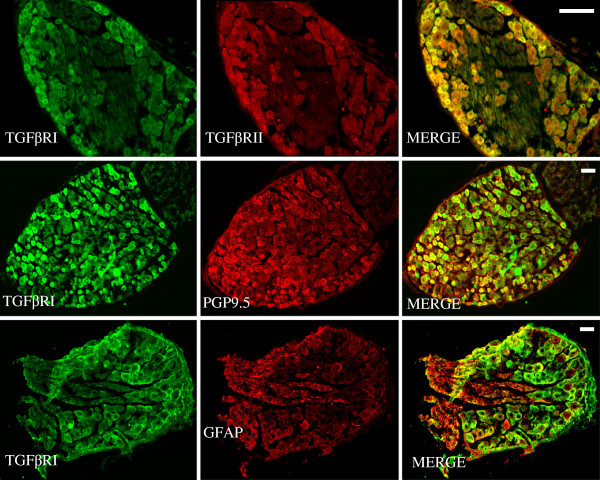
**TGFβRI expression in dorsal root ganglia neurons was analyzed by immunofluorescent staining on sections.** Left to right: Upper pannel- TGFβRI (green), TGFβRII (red) and a merged image; Middle pannel-TGFβRI (green), PGP9.5 (red) and a merged image; Bottom pannel-TGFβRI (green), GFAP (red) and a merged image. (Scale Bar = 75 μm).

TGFβ activation is mediated through two receptors (TGFβRI and TGFβRII) that work in series: TGFβRII is necessary for the initial binding of TGF and subsequent recruitment of the type I receptor and initiation of the signaling cascade. We confirmed previous reports of the expression of TGFβ receptors in DRG [5]. TGFβRI and TGFβRII were co-expressed in all neurons and a substantial proportion of glia in DRG using immunochemistry (Figure 1).

**Figure 2:**
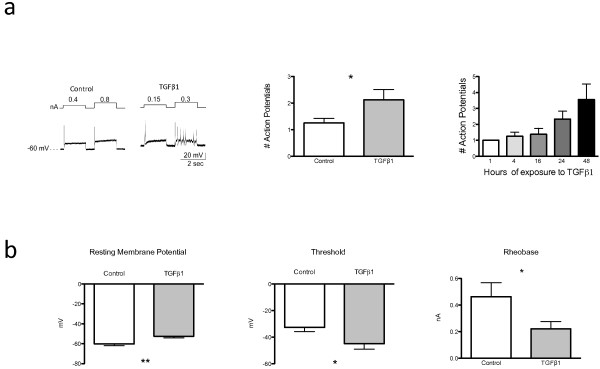
**a. *Left: *Representative tracing of action potentials displayed by DRG neurons in culture after 48 hours of exposure to TGFβ1, in response to current injections of 1x or 2x rheobase. ***Middle:* Bar graph showing the average number of action potentials evoked by 2x rheobase current injection after 48 hours of culture with TGFβ1 or control. *Right:*.Action potential frequency of DRG neurons in cultures increases with increasing duration of exposure to TGFβ1 (P =0.02 by ANOVA). **b**. Bar graphs showing electrophysiological changes in cultured DRG neurons after 48 hours of exposure to TGFβ1. *Left:* Resting membrane potential (n = 19 in each group); *Middle:* Voltage threshold for triggering an action potential (n = 19 in each group); *Right:* Rheobase (current required to trigger an action potential) (n = 20 in each group). *P <0.05. **P <0.01.

To assess the effect of TGFβ1 on neuronal excitability, we added TGFβ1 (10 ng/ml) to rat DRG cultures for 48 hours and then measured the electrophysiological properties of neurons (ranging in size from 15–30 μm) using whole-cell patch-clamp recording techniques. Under current clamp mode, in response to 2 x rheobase current injections, an approximately two-fold increase in evoked spikes were observed in the majority of neurons treated with TGFβ1 (Figure 2a), averaging 2.13 ± 0.52 spikes (n = 16 from 8 rats) as compared with 1.15 ± 0.26 in controls (n = 20 from 6 rats; P < 0.05). Examination of the time course of the TGFβ1 sensitization showed that it occurred at 24–48 hours of exposure but not earlier, suggesting that this effect is not due to direct effects on the membrane (Figure 2a, right panel). Other measures of excitability were also examined (Figure 2b): the resting membrane potential (RMP) was less negative in TGFβ1 treated neurons (−52.6 ± 2.28 mV; n = 19, 7 rats) as compared with control neurons ( −60.2 ± 2.45 mV; n = 19, 6 rats; P = 0.002). The voltage threshold for triggering an action potential was lower, i.e., nearer to the resting membrane potential in TGFβ1 treated neurons (−45 ± 14.96 mV; n = 14, 8 rats) as compared with control (−32.71 ± 3.36 mV; n = 14, 6 rats; P = 0.02). The rheobase (the minimal current pulse required for triggering an action potential) was also smaller in neurons exposed to TGFβ1 (0.22 ± 0.05 nA; n = 20, 7 rats) as compared with controls (0.46 ± 0.15 nA; n = 20, 6 rats; P =0.04).

**Figure 3:**
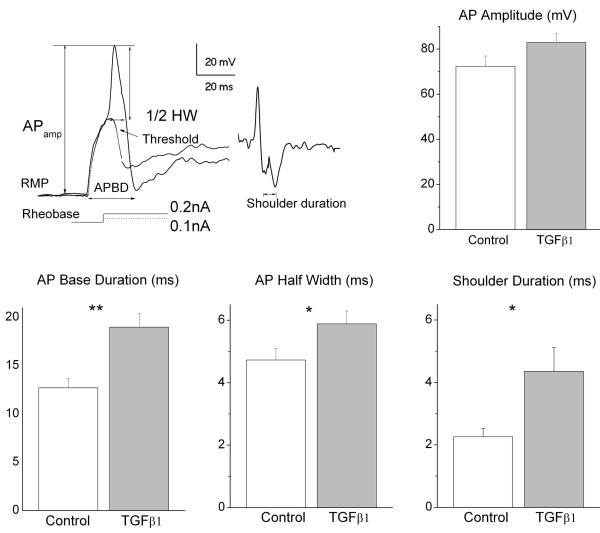
**Analysis of action potential after incubation with TGFβ1.** Top: *Left*, a representative action potential (AP) tracing (solid line) and threshold (dotted line) taken from a DRG neuron treated with TGFβ1. The single AP was evoked by a current injection at rheobase of 0.2 nA, and the parameters were measured for AP amplitude, threshold, Action potential durations at base (APBD) widths at half amplitude (AP ½ HW). The associated trace is a derivative of AP showing shoulder duration appeared on the falling phase of the AP. *Right*, AP amplitudes were not different in the two groups: 72.37 ± 4.5 mV in control (n = 19) and 82.94 mV in TGFβ1 group (n = 17; P = 0.11). Bottom: *Left*, APBD: controls = 12.7 ± 0.94 ms (n = 19) and TGFβ1 treated neurons = 18.97 ± 1.44 ms (n = 17). *Middle,* AP ½ HW: controls = 4.73 ± 0.37 ms (n = 19) and TGFβ1 treated neurons = 5.89 ± 0.41 ms (n = 17). *Right,* Shoulder durations: controls = 2.26 ± 0.26 ms (n = 19) and TGFβ1 treated neurons = 4.36 ± 0.76 ms (n = 17).*P <0.05,**P ≤0.01.

Analysis of action potentials (AP), as illustrated in Figure 3, revealed that TGFβ1 resulted in an increase in the AP base duration (18.97 ± 1.44 ms, n = 17 versus 12.7 ± 0.94 ms, n = 19, in controls) as well as in the half -width (5.89 ± 0.41 ms, n = 17 versus 4.73 ± 0.37 ms, n =19 in controls; P = 0.04). Action potential shoulder duration obtained from digital differentiation (dV/dt) was prominently broadened (4.36 ± 0.76 ms n = 17 versus 2.26 ± 0.26 in controls, n = 19; P = 0.01).

However, the amplitude of action potentials was similar in both TGFβ1-treated (82.94 ± 4.06 mV; n =17) and control neurons (72.37 ± 4.56 mV; n = 19; P = 0.1). Together these changes suggest a marked increase in membrane excitability in response to TGFβ1 treatment.

### TGFβ suppresses A-type potassium currents in sensory neurons

**Figure 4:**
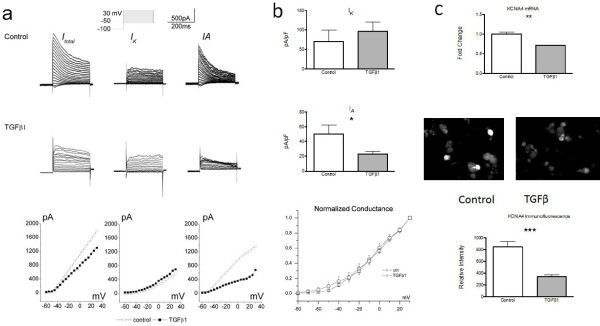
**a. Representative K+ currents in DRG neurons treated with TGFβ1.** Step depolarizations from −60 to +30 mV in 5-mV increments (duration = 400 ms, holding potential = −100 mV) were used to activate all Kv channels (Itotal) in cultured DRG neurons with or without exposure to TGFβ for 48 hours (left column, top two rows). Manipulating the holding potential to –50 mV with the same depolarization steps activated most of the sustained Kv channels but not IA channels (middle column, top two rows). Subtraction of IK from Itotal yields IA (right column, top two rows). The peak I-V curves for IA are shown in the bottom row. **b**. Quantification of IK (top) and IA (middle) currents in TGFβ1-treated and control neurons. TGFβ1 treatment resulted in a significant reduction in IA density at +30 mV (28.58 ± 3.958, n = 25 versus 68.67 ± 11.95 n = 17) but not in IK density. The bottom panels shows the normalized conductance (G/V relationship) for IA currents in control (n = 7) and TGFβ1 treated neurons (n = 10). **c**. Changes in KCNA4 expression in response to TGFβ1. Top: mRNA expression in DRG cultures treated with TGFβ1, expressed relative to GAPDH mRNA expression. Middle: Representative immunohistochemistry images from control and TGFβ1 treated cultures. Bottom: Bar graph showing relative intensity of cell fluorescence (averaged per high power field) showing significant decrease after 48 hours of exposure to TGFβ1 as compared with controls (854.3 ± 84.61, n = 11 fields versus 11348.3 ± 25.47, n = 8 fields). ** P < 0.01; ***P < 0.001.

We have previously determined that changes in voltage-gated potassium (Kv) currents are important in contributing to the excitability of DRG neurons in rats with chronic pancreatitis [10]. We therefore examined the possibility that TGFβ1 treatment affects Kv currents in DRG neurons, specifically focusing on the transient 'A-type' current (*I*_A_) and the 'sustained delayed rectifier type' (*I*_K_). TGFβ1 treatment resulted in a significant reduction in *I*_A_ density (23.16 ± 4.02 pA/pF, n = 10, 7 rats) as compared with controls (50.46 ± 12.15 pA/pF, n = 7, 5 rats, P = 0.02) (Figure 4b, middle). We also compared *I*_*A*_ conductance between two groups and observed no significant differences between TGFβ1 treated neurons and controls (Figure 4b, bottom). Moreover, TGFβ1 treatment did not result in a significant reduction of *I*_*K*_ density (Figure 4b, top), averaging 96.64 ± 22.93 pA/pF in TGFβ1 treated neurons (n = 10, 7 rats) versus 70.95 ± 29.28 pA/pF in controls (n = 7, 5 rats; P = 0.5). 

The molecular basis of *I*_A_ is incompletely understood, with the most commonly implicated Kv subunits being 1.4 (KCNA4) and 4.3 (KCND3) [11,12]. We therefore investigated the effects of TGFβ1 on the expression of these genes in sensory neurons using RT-PCR and found a significant decrease in KCNA4 mRNA levels (Figure 4c, top); while KCND3 (Kv4.3) mRNA levels were not significantly different (data not shown). Although the total number of immunopositive DRG cells did not differ between the treatment and control group (93% versus 97%), analysis of fluorescent intensity per cell showed a significant decrease in Kv1.4 fluorescence in neuronal cultures treated with TGFβ1 (Figure 4c, middle and bottom). Taken together, these results suggest that TGFβ1 modulates the regulation of KCNA4 gene expression in individual nociceptors.

### TGFβ1 induces pancreatic hyperalgesia in vivo

**Figure 5:**
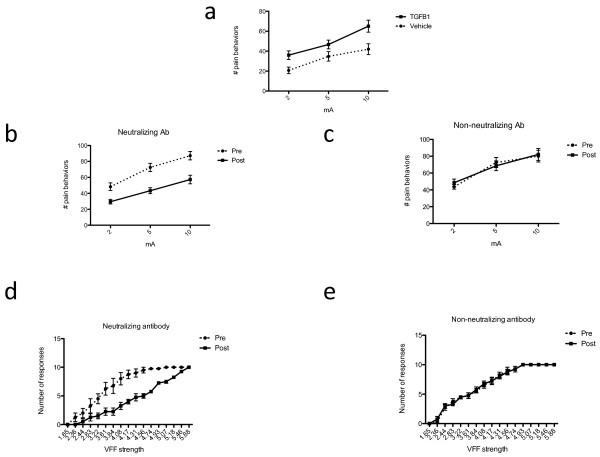
**a. Behavioral response (“pain behaviors”) to electrical stimulation of the pancreas 16–24 hours after intrapancreatic infusion of TGFβ1 or vehicle.** The graph shows the pooled results of two replicate experiments, each of which showed a statistically significant result when analyzed independently. Intrapancreatic TGFβ1 results in a significant upward shift of the stimulus response curve (P < 0.0001 for stimulus and P < 0.01 for TGFβ1 effect by two way ANOVA; n = 10 in the TGFβ1 group and n = 9 in the vehicle group). **b**, **c**. Attenuation of pancreatic hyperalgesia (as measured by the behavioral response to electrical stimulation) in a rat model of chronic pancreatitis by intraperitoneal injection of a neutralizing antibody to TGFβ1 (left panel) or a non-neutralizing antibody also directed against TGFβ1 (right panel). Baseline responses were obtained and then repeated one week after administration of the antibody. Treatment with the neutralizing antibody resulted in a marked attenuation of the behavioral response to electrical stimulation of the pancreas as compared to baseline (P < 0.0001 for both stimulus and treatment; n = 9) whereas administration of the non-neutralizing antibody resulted in no change in the response (n = 9). **d**, **e**. Attenuation of referred somatic hyperalgesia, as measured by Von Frey filament (VFF) testing. Treatment with the neutralizing antibody resulted in a decrease in the response frequencies to abdominal wall probing as compared to baseline (P < 0.0001 for both stimulus and treatment) whereas administration of the non-neutralizing antibody resulted in no change in the response.

We first infused TGFβ1 (400 ng in 400 μL) into the pancreas of rats and 24 hours later measured the subsequent behavioral response to noxious electrical stimulation of the pancreas, an established method for testing nociception in this organ [13,14]. Figure 5a shows the pooled results of two replicate experiments, each of which also showed a statistically significant change when analyzed independently. Intrapancreatic TGFβ1 infusion results in a significant upward shift of the stimulus response curve (P < 0.0001 for both stimulus-induced response and TGFβ1 effect by two-way repeated measures ANOVA; n = 10 in the TGFβ1 group and n = 9 in the vehicle group). Histopathological examination did not reveal any differences in pancreatic morphology between the two groups.

### TGFβ1 contributes to pain behavior in a rat model of chronic pancreatitis

We next assessed the effects of neutralization of TGFβ1 on pain behavior in a rat model of chronic pancreatitis using a neutralizing antibody which has been shown to be effective in antagonizing TGFβ1 effects lasting up to six weeks or more [15]. Control rats were given the same dose of another antibody against TGFβ1 but without neutralizing properties. Rats underwent testing for pain behavior in response to electrical stimulation at baseline and one week after treatment. Figures 5b and c show the pooled results of two replicate experiments (each of which also showed a statistically significant change when analyzed independently). Those receiving the neutralizing anti-TGFβ1 antibody displayed a significant reduction in pain behaviors in response to electrical stimulation (Figure 5b; two-way repeated measures ANOVA: stimulus effect, P <0.0001; treatment effect, P <0.0001; n = 9). Applying a Bonferroni post-hoc test, this effect is significant at all three intensities of electrical stimulation. By contrast, the non- neutralizing antibody had no effect on the responses to electrical stimulation (Figure 5c; two-way repeated measures ANOVA: stimulus effect, P = 0.0003; treatment effect, P = 0.70; n = 9).

In a subset of these rats (n = 4 in each group), we also examined referred somatic hyperalgesia using von Frey filament (VFF) testing as previously described in this model [15] (Figures 5d and e). Overall, the response frequencies of rats treated with the neutralizing antibody were significantly lower compared to pretreatment baseline, with the stimulus–response curve shifting lower (two-way repeated measures ANOVA: stimulus effect, P <0.0001; treatment effect, P <0.0001). On the other hand, rats treated with the non-neutralizing antibody did not show any change in their response frequencies (stimulus effect, P < 0.0001; treatment effect, P = 0.84).

**Figure 6:**
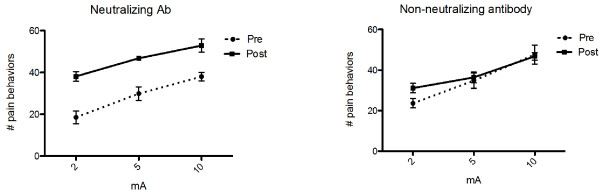
**Effect of TGFβ neutralization on pancreatic nociception in naïve rats.** Intraperitoneal injection of a neutralizing antibody to TGFβ1 (left panel) or a non-neutralizing antibody also directed against TGFβ1 (right panel) was administered as described in the text. Nocifensive behavior was measured in response to to electrical stimulation. The graphs show the pooled results of two replicate experiments, each of which showed a statistically significant result when analyzed independently. Baseline responses were obtained and then repeated one week after administration of the antibody. Treatment with the neutralizing antibody resulted in a enhancement of the behavioral response to electrical stimulation of the pancreas compared to baseline (P < 0.0001 for both stimulus and treatment; n = 8) whereas administration of the non-neutralizing antibody resulted in no changes in the response (n = 8).

Neutralization of TGFβ1 had no significant effect on histological signs of inflammation in pancreatic specimens. Unexpectedly, by contrast to rats with chronic pancreatitis, TGF neutralization resulted in hyperalgesia to electrical stimulation in naïve rats, as shown in Figure 6 (n = 8, P < 0.0001 for both stimulation and treatment effect), whereas the non-neutralizing antibody had no effect (n = 8, P <0.0001 for stimulation, P =0.31 for treatment effect).

## Discussion

Ongoing tissue injury and inflammation initiate a cascade of events resulting in peripheral sensitization i.e. enhancement of the responsiveness of primary afferent neurons (nociceptors), whose bodies lie in dorsal root ganglia (DRG) and whose central ends synapse with second order neurons in the spinal cord. Sensitized nociceptors display increased spontaneous activity as well as increased responsiveness to both noxious and non-noxious stimulation. While post-translational changes in key ion channels and receptors underlie the immediate/acute phase of sensitization, sustained/chronic peripheral sensitization is also accompanied by neuroplastic transcriptional events induced by biologically active components in the environment.

Although TGFβ is prominent in this milieu and its receptors are expressed by DRG neurons [5], its participation in sensitization of the primary nociceptor (peripheral sensitization) has received little attention. There is some evidence that TGF may participate in the *central* processing of pain signals. Intrathecal infusion of anti-TGF β antibody suppresses glial activation and spinal inflammation and attenuates neuropathic pain induced by nerve injury in rats [16]. An analgesic role for TGFβ in the CNS also appears to be indirectly supported by the attenuation of acute and chronic pain in mice lacking BAMBI (Bone Morphogenetic Protein and Activin Membrane-Bound Inhibitor), a pseudoreceptor that binds TGFβ and negatively modulates its signaling [17]. In the CNS, the analgesic effects of TGFβ may be attributed to the suppression of glial activation and spinal inflammation, both of which are associated with pain [18].

On the other hand, TGFβ can also have potentially pro-nociceptive effects on nociceptors: human TGFβ causes increased firing of Aplysia nociceptive neurons, a decrease in their threshold, long-term synaptic facilitation and a reduction in synaptic depression [19-22]. Further, activin, a member of the TGF family, induces neuropeptide expression in nociceptors and sensitization of the vanilloid receptor, TRPV1 along with hyperalgesia in rats [23-25].

Thus a role for TGFβ in inflammatory pain, particularly with respect to direct effects on nociceptors in peripheral tissues, has yet to be established conclusively. In this paper we provide the first evidence for a convincing role of TGFβ in peripheral sensitization, from both *in vitro* and *in vivo* experiments. DRG neurons in culture show a robust increase in excitablity after incubation with TGFβ1, with significant changes in several electrophysiological attributes. These changes are consistent with what we have previously described in this model of chronic pancreatitis, with changes in resting membrane potential, decreased rheobase, and increased number of spontaneous and evoked action potentials [13]. Further, we found an increase in action potential duration, similar to what has been reported for cAMP- and capsaicin-induced broadening of the AP, which was attributed to a decrease in voltage-gated potassium (Kv) currents [26].

In order to understand the underlying basis for the changes observed in this study, we focused on the effects of TGFβ1 on Kv currents, which we have previously shown to be significantly downregulated in our model of chronic pancreatitis [10]. We found that TGFβ1 can cause downregulation in *I*_*A*_ currrents, also similar to what we have previously observed in pancreatic nociceptors of rats with chronic pancreatitis. Further, this change in current was associated with the downregulation of expression of a specific gene subserving these currents, KCNA4, but not KCND3, both of which can encode these currents in neurons [11,12]. KCNA4 may be the dominant Kv1 alpha subunit expressed in TRPV1 expressing smaller diameter neurons in the DRG [11], and changes in KCNA4 have been described in other models of pain including cystitis [27-29]. However, TGFβ1 may have effects on other ion channels and signaling pathways that may contribute to increased excitability and sensitization, and these posibilities need to be examined in future studies. Further, given that DRG cultures contain a mixture of glia and neurons, we cannot exclude the possibility that some of the effects on neurons are indirect, and mediated by glia in response to TGFβ1.

Regardless of the *in vitro* mechanism, TGFβ1 infusion into the normal pancreas also induces abdominal hyperalgesia due to pancreatic stimulation. Conversely, TGFβ1 antagonism can attenuate hypersensitivity and hyperalgesia in chronic pancreatitis, a painful inflammatory condition. These studies do not imply that TGFβ is the sole or even dominant contributor to nociceptive sensitization in chronic pancreatitis, where many other factors, such as NGF may also play a role [30,31]. Surprisingly, TGFβ antagonism caused hyperalgesia to noxious stimulation in naïve rats, suggesting that endogenous TGF plays a tonic modulatory effect in nociception signal processing and that the effects of TGF on nociception are likely to be complex and bimodal, as has been described for other biological consequences of TGF neutralization [32]. Thus, under normal physiological conditions, TGFβ may be required for maintaining sensory neurons in a healthy state whereas in chronic inflammation, excessive levels may cause sensitization. The physiological role of TGFβ in maintaining nociceptor sensitivity requires further studies and these are currently underway in our laboratory. It also appears that TGFβ may indeed have dual and seemingly opposing roles in nociception in the peripheral versus central nervous systems, similar to what has been reported for other peptides such as nociceptin [33]. Our studies do not exclude the possibility that TGFβ may exert effects on other important ion channels in nociceptors nor do they pinpoint the signaling pathways involved in the observed changes in excitability. Finally, isomers other than TGFβ1, used in this study, may have different effects on nociception. There are at least three different TGFβ isomers (1, 2 and 3) with varying degrees of tissue specific expression: all share a common signaling pathway that involves both the canonical SMAD pathway as well as non-canonical (e.g. involving MAP kinases) [34,35]. Further molecular studies to understand the mechanism by which TGFβ exerts these effects are underway.

## Conclusions

We have shown that TGFβ1 can result in peripheral sensitization and contribute to the enhanced nociception that accompanies chronic inflammation. Further, our results suggest that this effect may involve the suppression of IA currents, providing a mechanistic explanation for the increased neuronal excitability. TGFβ1 is therefore an important modulator of peripheral sensitization of nociceptive neurons. As such, further understanding of the role of the pathway in nociceptor neurons may provide insight into new therapeutic targets for the treatment of such conditions.

## Methods

All experiments were approved by the Institutional Animal Care and Use Committee at Stanford University in accordance with the guidelines of the International Association for the Study of Pain. Male Sprague–Dawley rats (Harlan, Indianapolis, IN), weighing between 250-280 g, were used in the experiments.

### Dorsal root ganglia (DRG) neuron culture

After decapitation, thoracic and lumbar DRGs were dissected out and transferred to ice-cold Minimal Essential Medium (Gibco, Grand Island, NY) supplemented with penicillin-streptomycin (2X, Gibco). After trimming the axons and connective tissue, ganglia were transferred into Hank’s Balanced Salt Solution containing 5 mg/ml collagenase (Type II, Worthington, Lakewood, NJ), and incubated for three hours at 5% CO_2_-95% O_2_ at 37°C. A single cell supension was subsequently obtained by repeated trituration through flame-polished glass pipettes and centrifuged at 50×g for 10 minutes. Single cells were resuspended in neurobasal media (Gibco) supplemented with albumin solution (0.7%, Sigma, St. Louis, MO), penicillin-streptomycin (2X), B27 with retinoic acid (2X, Invitrogen, Carlsbad, CA), β-mercaptoethanol (0.11 mM, Gibco), mouse nerve growth factor (40 ng/ml, Promega, Madison, WI) and L-glutamine (2X, Gibco) and plated onto poly-l-ornithine (Sigma) coated coverslips.

Recombinant TGFβ1 (Calbiochem, Gibbstown, NJ) was applied to the culture media in a concentration of 10 ng/ml. During culture in 36.5°C 5% CO_2_ incubator, the culture media (with and without TGFβ1) were refreshed every 24 hours.

### Electrophysiology

Whole-cell voltage patch-clamp recordings were conducted at room temperature (22–23°C) on the stage of an inverted phase contrast microscope (Nikon Inc., Melville, NY). The recording pipettes were pulled from borosilicate glass to give resistances of 2–6 MΩ. Data were acquired with Digidata interface 1200 series, and pClamp software version 9.1 (Molecular Devices, Sunnyvale, California). The concentration in the pipette solution were as follows (in mM): K gluconate (115), KCl (25), NaCl (5), HEPES (10), CaCl (1), EGTA (1.12) and ATP-Mg(2), pH was adjusted to 7.3–7.4 using KOH (280–300 mOsm). The cells were bathed in modified Tyrode saline consisting of (in mM): NaCl (135), KCl (5.4), MgCl_2_ (1), CaCl_2_ (2) NaH_2_PO_4_ (0.1) HEPES (10) glucose (10), with pH adjusted to 7.3-7.4 using NaOH (300–320 mOsm). In experiments that required eliminating Na^+^ current, [Na^+^]_o_ was substituted by equimolar choline.

Prior to patch clamping a cell, the amplifier (Axopatch 200B, Molecular Devices, Sunnyvale, USA) was zeroed so that any junction potential was balanced by an offset potential. High resistance (Gigaohm) seals were formed between the recording electrode and cell membrane and ruptured by suction using standard patch clamp recording methods. Action potentials were recorded in mode of I-clamp after obtaining a stablized membrane potential setting at I = 0. 2-step current stimulation pulses were injected for a length of 1.8 sec at 1x and 2x rheobase with an interval of 600 ms. Current pulses were repeated in a range of 0.01 to 1 nA steps until an AP was elicited. Action potential threshold was determined upon the voltage extent before upstroke. Currents were recorded under the mode of V-clamp and the current signals were recorded to disk for off-line analysis using pClampfit and Origin 7. Results were expressed as means ± SE, n = number of cells.

### Immunohistochemistry of DRG sections

DRGs (T9–13) were removed and postfixed for 4 hours in 4% paraformaldehyde and cryoprotected overnight in 30% sucrose in PBS. Tissue was embedded in optimal cutting temperature (OCT) and 10 μm frozen sections were prepared. Sections were blocked and permeabilized for 1 h at room temperature with PBS containing 0.3% Triton X-100 and 10% normal goat serum and incubated overnight at 4°C with primary antibodies diluted in PBS containing 1.5% normal goat serum. The following antibodies were used: mouse monoclonal antibody TGFβR I (1:100; ab27969), rabbit polyclonal TGFβR II (1:100; ab66045; abcam, Cambridge, MA, USA), Polyclonal Rabbit Anti-PGP 9.5 (1:400; Dako), and αGFAP (rabbit 1:400; Dako, Carpinteria, CA). After washing with PBS, secondary goat antibodies anti-mouse IgG488 and anti-rabbit IgG 594 (Invitrogen, Carlsbad, CA, U.S.A.) were added to the preparations at 1:200 dilution. Sections were rinsed with PBS 15 min ×3 times and viewed under a fluorescent microscope (Nikon Eclipse E600, Japan) with an excitation wavelength appropriate for 488 and 594.

### Immunohistochemistry and quantification of fluorescence for KCNA4 expression

DRG cultures with and without TGFβ1 treatment at 48 hours were fixed in 4% paraformaldehyde (PFA) for 30 minutes. Nonspecific antibody binding was blocked by incubation with 8% normal horse serum plus 1% bovine serum albumin for 1 hour. The preparation was then incubated with monoclonal mouse anti-Kv1.4(1:200; NeuroMab, UC Davis, CA, U.S.A.) overnight at 4°C plus 1 hour at room temperature. Secondary goat antibody anti-mouse IgG 488 (Invitrogen, Carlsbad, CA, U.S.A.) was added to the preparations at 1:200 dilution. Each step was rinsed with PBS for 15 min × 3 times. Staining was examined with a fluorescent microscope (Nikon Eclipse E600, Japan) with an excitation wavelength appropriate for 488. All procedures were done under the same conditions including staining and scanning. Quantification of Kv1.4 expression was performed using the public domain NIH ImageJ program ( http://rsb.info.nih.gov/nih-image/). The area of immunopositive cells was determined by threshold with subtraction of background noise and then expressed as mean of fluorescent intensity per high power field.

### RT-PCR of KCNA4 gene expression

RNA was extracted from DRG cultures with and without TGFβ1 treatment at 48 hours as described above. cDNA was made from 100 ng of total RNA prior to being pre-amplified for 14 cycles in the presence of various taqman primers obtained from ABI (Foster City, CA). Fold change was determined by the delta delta Ct method after normalizing to GAPDH and expressed relative to the mean value of the control group.

### Intrapancreatic infusion of TGFβ1

Under anesthetization with ketamine/xylazine, the peritoneum was incised to expose the duodenum and the duodenal loop was pulled out. The pancreatic duct entering the duodenum was identified under dissecting microscope and a small nick was made into the duct. A 30-gauge needle with polyethelene 10 tubing (Becton Dickinson and Company, Franklin Lakes, NJ) was guided into the pancreatic duct whilst the common bile duct was loosely ligated at both ends. 400 μl of a 10% ethanol in phosphate buffered saline containing 400 ng of TGFβ1 (R&D Systems, Minneapolis, MD) or vehicle alone was injected into the pancreatic duct. The tubing was carefully removed and bile flow from the liver into the duodenum was re-established. A pair of electrodes was carefully sutured into the pancreatic tissue as described below under “evaluation of pain behavior”. After removing the tube, the abdominal cavity was closed with sutures and rats were allowed to recover. Pain behavior was assessed in both groups of rats 24 hours post TGFβ1 infusion.

### Induction of chronic pancreatitis in rats

The pancreatic duct was accessed as described above and 0.5 ml of 6 mg/ml solution of trinitrobenzene sulfonic acid (TNBS), in 10% ethanol in PBS (pH 7.4) was infused over a period of 2 to 5 minutes. Needle and tubing were then removed, the abdominal cavity was closed with sutures and rats were allowed to recover. Rats underwent further intervention at three weeks when a robust chronic pancreatitis has developed, as described previously by us [13,36].

### Evaluation of pain behavior

At the time of surgery for intraductal infusion of TNBS, a pair of electrodes was attached to the pancreas and externalized behind the head, as previously described [13,36], and the rats were allowed to recover. At specified times rats were given successive applications of current at 2, 5 and 10 mA for 5 min at a 10-min interval between stimulation periods. The number of nocifensive pain behaviors during the stimulation period was measured. Pain behavioral responses consisted of stretching, licking of the limbs and abdomen, contraction of abdominal wall muscles and extension of the hind limbs as previously described [13,36]. All the tests were performed by an observer blinded to the treatment.

### Blockade of TGFβ1

Three weeks after infusion of TNBS, we injected a neutralizing antibody (MAB 240, R&D Systems, Minneapolis, MD) in a single dose of 1 mg/kg intraperitoneally to a group of rats. Control rats received the same dose of another antibody against TGFβ1 which did not have neutralizing properties (MAB 2401, R&D Systems). At baseline and one week after the injection, rats underwent testing for pain behavior. At this dose, the neutralizing antibody has been shown to be effective in antagonizing TGFβ1 effects up to six weeks or more [15].

### Statistical analysis

Results were expressed as means ± SEM, with *n* being the number of cells. The paired Student's *t*-test was used to evaluate differences between mean values of two groups. For multiple groups, ANOVA was used. *P* values of ≤0.05 were considered to indicate a statistically significant difference. I_A_ current conductance was determined according to the formula G = I/(V_t_ − V_rev_) where G is the conductance, V_t_ is the test potential at which current is measured, and V_rev_ is the reversal potential.

## Abbreviations

TGFβ: Transforming growth factor beta; Kv: Voltage-gated potassium channel; KCN: Potassium channel; *I*_A_: Transient 'A-type' potassium current; *I*_K_: Sustained delayed rectifier type'.

## Competing interests

Stanford University has applied for a patent on the use of TGFβ antagonists for the treatment of pain.

## Authors’ contributions

YZ, TC, MS, LL, CL, KM. BZ, SX: Acquisition of data, editing of manuscript. RP: Analysis of pathological findings. PJP: Conception, planning and oversight of experiments; interpretation of data; writing and finalizing manuscript. All authors read and approved the final manuscript.
